# University College, Bristol. Faculty of Medicine

**Published:** 1893-12

**Authors:** 


### University College, Bristol.

### FACULTY OF MEDICINE.

Mr. Albert Fry, Chairman of the Council of the Bristol University College, presided on October 20th at a meeting held in the Theatre of the Museum and Library, when the Right Honourable Sir Edward Fry, LL.D., F.R.S., distributed the prizes to the successful students of the Medical School, which is now incorporated with the College.

The Dean of the Faculty (Dr. Markham Skerritt) read the report and list of prizes. The report contained the following:—

The Faculty of Medicine have much pleasure in making special record of two events which have occurred during the past year, which are, in their opinion, of great importance to the medical department of the College, and destined to have a marked influence upon its future. One is the public opening of the long-needed new buildings on the 18th of last November by Sir Andrew Clark, president of the Royal College of Physicians. In the handsome structure now at their disposal the Faculty have ample provision for carrying on with efficiency the work of the department in its various aspects. In addition to the portion more especially devoted to teaching purposes, a large hall has been provided for use as a library, and the Council have made arrangements for the centralisation here of all the more important medical libraries at present in existence in the neighbourhood. They are consequently already in possession of a most extensive collection of works, comprising not only the medical library of the College, but also those of the Bristol Medico-Chirurgical Society, the Bristol Royal Infirmary, and the Bristol General Hospital; while the medical works formerly belonging to the Bristol Museum and Library will also be transferred to the Medical Library of the College. There will thus be formed an extensive reference library, the value of which, not only to the students of this College, but also to the members of the medical profession of the West of England, cannot be over-estimated. The second event to which reference has been made is the incorporation of the Bristol Medical School with the College. In existence since 1828, the Medical School was affiliated to the College at the foundation of the latter, continuing to have a governing body independent of the Council of the College. As it was the opinion of both the Council of the College and the Faculty of the Medical School that a closer union would be a source of strength to both institutions, negotiations were entered into whereby on the 12th April of the present year the Bristol Medical School ceased to have an independent existence, and became merged in the College as its Faculty of Medicine. The Faculty have to congratulate themselves, and their College, on the appointment of a Professor of Anatomy who is prepared to give his whole time to the work of teaching. Dr. Edward Fawcett has already distinguished himself in the teaching of Anatomy at the Yorkshire College, and he has entered on his duties at Bristol with every prospect of continued success.

After some observations addressed to the successful and unsuccessful students respectively, in the course of which he observed that the capacity to win prizes was by no means an accurate measure of the capacity to succeed in life, Sir EDWARD FRY said:

I confess that I never take part in gatherings of this description without a certain amount of hesitation and doubt. I am fully alive to the value of the capacity of rapidly acquiring a great mass of information and of reproducing it in a clear and concise form, which is promoted by competitive examinations; and I know that the extension of such examinations has been coincident with a great advance in education in this country in almost all branches of learning, and not least in those which are connected with medical science. But nevertheless I feel strongly the evils which are incident to competitive examinations.

By some mysterious quality of our nature the object with which knowledge is acquired affects the knowledge itself: the purpose which is present in the will operates in some way on the intellect; so that on the one hand, information acquired for the purpose of being used on some particular occasion is wont to vanish from the mind so soon as that occasion is passed; and on the other hand, knowledge acquired for its own sake—for the sake of really knowing—is fixed into the mind as it were by a mordant.

So soon as the looked-for examination is over, the mind is apt to suffer a severe collapse, and this occurs not only on particular occasions, but often affects the whole habit of the intellect; and thus many men who have had a conspicuous success in the schools, so soon as they have come to the end of their educational curriculum, feel as if all motive for learning were withdrawn, and live thereafter as men with no curiosity to satisfy, with little or no real thirst for knowledge. They find too late the fact that they have put the desire for success in examinations, which is a temporary and limited motive, into the place of the desire to know, which is a permanent and infinite motive for the mind.

I am aware that examinations are imposed upon you, and I therefore allude to these incidental evils in the hope that you may be on your guard against them, and may cultivate as far as possible that eagerness for truth for its own sake which will go far to counteract them.

You have the great good fortune to live in a period of marked and rapid advance in science, and not least in the biological sciences with which you are chiefly concerned. So great has been this advance, so great the extension of the means of enquiry and research, that you are thereby exposed to the dangers and the embarrassment of an excess of wealth.

There is another difficulty which lies in your way to which I will venture to allude, and in doing which I shall, I believe, have the concurrence of many who are sitting near me, and who are far more competent to deal with the subject than I can be. Of medical literature I will not say anything; but of some scientific writing (I say some, for I know that there are great exceptions) I will dare to say that it appears to me to be justly open to much criticism. In the first place, the productions to which I refer are devoid of style—by which I do not mean that they are wanting in rhetoric, but that they are produced without any serious and deliberate effort at clearness and simplicity. I know that it is often a wearisome task to put order and light into the statement of complicated and subtle facts; but it is a task which the reader has, in my humble opinion, a right to demand from the writer. Instead of an orderly marshalling of facts and arguments, how often do we get presented to us amorphous masses of facts and amorphous masses of authority, and the reader is left to think the matter out for himself as best he can, and to restate it so as to be intelligible to himself.

Again. we are infected by an error—an old sin from which we sometimes thought that we had got away: I mean the idolatry of great names. How often is our attention occupied, not by the facts of the case, but by some discussion as to what Mr. Darwin thought or said or meant on some particular point? This is, I have said, an old sin. So it was with the followers of Pythagoras when the question was to be decided by the αὐτὸς ἔφη. So it was with the followers of Aristotle. So in later times with the followers of Linnæus. These great men so impressed the minds of their followers that they seemed to them to have reached the utmost bounds—the *ultima Thule* of knowledge; and they followed them by adopting their facts and their conclusions, instead of by adopting their methods and their spirit, and so rendering to them the truest of homage.

Another count in my indictment against some of our modern scientific writing is based on another old error that is still amongst us: I mean the habit of introducing some metaphysical term or some abstract word which expresses only an aggregate of facts as if it were a true cause; and thus we get metaphysical causes assigned for physical effects, or a fact accounted for by reference to some word which is itself only a statement of the very fact in question and a number of other kindred facts.

Again, I venture to think that the logic of a great deal of scientific writing stands in need of correction. So small a part of the propositions with which we have to deal in life and in the sciences of observation are absolutely and apodictically certain, and so large a part of them are probable and no more, that the logic of probabilities appears to me to deserve more attention than it sometimes receives. Suppose that it is probable that A is B; and if so, it is probable that C is D; and if so, it is probable that E is F; the odds may well be against the last of these propositions, because, as is easily seen, the probability of the last of a series of dependent probable propositions is much less than the probability of any one of these propositions; whereas in the case of independent and coinciding probabilities, the probability of the conclusion is greater than that of any one of the grouuds of inference. I sometimes venture to doubt whether these considerations receive due attention.[1]

Again, much of the writing to which I refer seems to me bad for want of mental suspension. Every wise man must often feel that there are matters on which he has not enough information to form a conclusion—matters in respect of which he ought to say to himself: "I do not know." And yet we are not unfrequently invited by scientific writers to accept some conclusion, not because it is proved, but because no better can be offered.

Thus it seems to me that there are difficulties which beset a student of science in the present day, arising partly from the rapid growth of science and partly from the method of its presentation. How are you to grapple with these difficulties? I answer in one word—by method. Cultivate a rigorous analysis of all that you learn, a thorough and careful apprehension of what you study, a habit of being able to get to

1 If there be three propositions, each of which has 6 to 4 in its favour, and of these propositions the second depends on the first in such sort that it does not arise unless the first be true, and the third depends in like manner on the second, the odds will be 98 to 27 against the truth of the last proposition. If, on the other hand, the three probabilities (each of 6 to 4) be independent and all lead to the same conclusion, the odds in favour of that conclusion will be 117 to 8.

the bottom of your subject. Such a habit will make you very slow sometimes in your apparent progress; but your pace will increase, and you will find that the clear light you have gained on some small area of knowledge will spread wider and wider as you go forward. Be careful to know the limits of your knowledge; for, depend upon it, that whilst the best thing is to know, the next best is to know that you do not know. Cultivate an absolute sincerity with yourself in this matter, and never pretend to yourselves, nor to anyone else—not even to a patient—to know what you do not know. I am not saying a word against half-knowledge; rather I would say cultivate it, for it is impossible that you can thoroughly know the whole area open to you: and it is a great thing to know something about subjects which you have not thoroughly studied, a great thing to know so much of the means of knowledge on many subjects as that when the need arises you may be able to pursue the thread. But take care to draw, strong and clear to yourselves, the line between a thorough knowledge and a half-knowledge.

Every profession has a tendency towards a pedantry of its own, towards a preference of technical to popular language. The rule on which one should act in this matter I conceive to be this: that when the technical word has a more precise and exact meaning than the popular one, then it should be used freely and courageously. But when the popular and the technical expressions have an equal fulness and exactitude of meaning, there the popular language should be preferred. There may be reasons for it; but I never quite understand why in some people's mouths a bleeding is always a hemorrhage, a bruise a contusion, a gathering an abscess, and a cut an incised wound. I know that juries are often enough puzzled by language which might be more simple, without, I think, much injury to the precision required.[2]

In the pursuit of medicine and surgery, and in those natural sciences which are ancillary to them, you have an infinite field for study and research. And, believe me, you will find the pursuit of knowledge its own great reward. But with you there is not merely this to draw you on; there is the sense of duty to urge you forward. For you are placed under a strong obligation by the profession which you have undertaken, to pursue knowledge; and by that sense of duty your love of knowledge should be interpenetrated and as it were fused to a white heat.

Gentlemen, the profession which you have chosen is a noble one: it has an ancient and illustrious history, a longer continuous story, I believe, than can be shown by any other branch of applied science; it will give ample scope and verge enough for the cultivation of your intellectual powers—for the faculties of observation, of reason, of thought; it will afford abundant exercise for all the best and highest parts of your moral nature, for kindness and beneficence, for self-denial, for self-sacrifice. It is noble above all things, because its object is to lessen the sufferings of humanity. Such a profession deserves to be followed worthily and nobly; and in the hope that you will so pursue it, I wish to you one and all, alike in your pupilage and in your practice, a hearty God-speed.

2 When I was on circuit some years ago, I think at Chester, a case came before my brother judge in which a witness was absent, and his son, a little boy, was called to prove his father's illness; and so, to let in the deposition he had made, "Well, my boy," said the examining counsel, "has the doctor seen your father?" "Yes," said the boy, "he have been, and says that father has a confusion in his abominable parts that will most likely lead to an absence."

